# Using Large-Scale Statistical Chinese Brain Template (Chinese2020) in Popular Neuroimage Analysis Toolkits

**DOI:** 10.3389/fnhum.2017.00414

**Published:** 2017-08-17

**Authors:** Lin Shi, Peipeng Liang, Yishan Luo, Kai Liu, Vincent C. T. Mok, Winnie C. W. Chu, Defeng Wang, Kuncheng Li

**Affiliations:** ^1^Department of Medicine and Therapeutics, The Chinese University of Hong Kong Shatin, Hong Kong; ^2^Department of Radiology, Xuanwu Hospital, Capital Medical University Beijing, China; ^3^Beijing Key Laboratory of MRI and Brain Informatics Beijing, China; ^4^Research Center for Medical Image Computing, Department of Imaging and Interventional Radiology, The Chinese University of Hong Kong Shatin, Hong Kong

**Keywords:** standard brain template, Chinese2020, SPM, MRI imaging, fMRI analysis

## Abstract

Given that the morphology of Chinese brains statistically differs from that of Caucasian, there is an urgent need to develop a Chinese brain template for neuroimaging studies in Chinese populations. Based on a multi-center dataset, we developed a statistical Chinese brain template, named as Chinese2020 (Liang et al., 2015). This new Chinese brain atlas has been validated in brain normalization and segmentation for anatomical Magnetic Resonance Imaging (MRI) studies, and is publicly available at http://www.chinese-brain-atlases.org/. In our previous study, we have demonstrated this Chinese atlas showed higher accuracy in segmentation and relatively smaller shape deformations during registration. Because the spatial normalization of functional images is mainly based on the segmentation and normalization of anatomical image, the population-specific brain atlas should also be more appropriate for functional studies involving Chinese populations. The aim of this technology report is to validate the performance of Chinsese2020 template in functional neuroimaging studies, and demonstrated that for Chinese population studies, the use of the Chinese2010 template produces more valid results. The steps of how to use the Chinese2020 template in SPM software were given in details in this technology report, and based on an example of finger tapping fMRI study, this technology report demonstrated the Chinese2020 template could improve the performance of the neuroimaging analysis of Chinese populations.

## Introduction

The human brain imaging techniques, such as Positron Emission Tomography (PET), Magnetic Resonance Imaging (MRI), and Magnetic Electrical Magnetoencephalography (MEG), have shown great potential to investigate the structural, functional and metabolic changes of the brain in both diseased and healthy states. Since the structure of human brain is variable across individuals, as well as gender, age and race (Kovalev et al., 2003; Brickman et al., 2008; Chee et al., 2011), it is crucial to map them into a standard template or atlas, so as to make the brains comparable between subjects/groups. To date, several human brain templates, such as the Talairach and Tournoux (1988) template^1^, the Montreal Neurological Institute (MNI305) template (Evans et al., 1993) and the International Consortium for Brain Mapping (ICBM152) template (Mazziotta et al., 2001) have been proposed and widely used in previous studies (Evans et al., 2012). However, the Talairach and Tournoux (1988) template was based on postmortem sections of a 60-year-old French female, and the MNI and ICBM template were developed based on western subjects; few brain templates were in fact built based on Chinese populations. With the fact that the morphology of east Asian brains statistically differs from that of Caucasian, e.g., east Asian brains are smaller in length and height than those of the Caucasians, but similar in width (Zilles et al., 2001; Lee et al., 2005; Tang et al., 2010), some bias may be introduced when we normalize the Chinese brain to the Caucasians template.

In recent years, some statistical brain templates were constructed based on the MRI data of some east Asian populations, such as Japanese atlas (with 56 subjects; Zilles et al., 2001), Korean atlas (with 78 subjects; Lee et al., 2005), and Chinese atlas (with 56 subjects; Tang et al., 2010). However, these templates were always limited by the small sample sizes of the population and were not widely used as a common space for the Asian-specific brain analysis.

Recently, in a controlled study using consistent MRI acquisition protocol and over 2000 subjects across multiple centers covering the major regions of China, we have developed a statistical Chinese brain template, named as Chinese2020 (Liang et al., 2015). This template has been demonstrated to offer higher representativeness of Chinese populations and could achieve better performance in morphology analysis of the Chinese brain (Liang et al., 2015). Besides the morphology analysis, this Chinese2020 template could also be used to improve the performance of brain functional analysis in Chinese population. In this technology report, we would like to give a detailed guideline on how to use the Chinese2020 template in the context of popular neuroimaging analysis toolkits (with SPM as an example) and demonstrate it could improve the performance of neuroimaging studies of Chinese population compared to the Caucasian templates.

## Materials and Methods

### Overview of Chinese2020

Chinese2020 is a statistical brain atlas based on a multi-center high quality MRI dataset of 2020 Chinese adults (18–76 years old). This is a new Chinese brain standard spatial template and its corresponding coordinate system and brain atlas labeling were further defined (Liang et al., 2015). This new Chinese brain atlas has been validated in brain normalization and segmentation, and is now publicly available at http://www.chinese-brain-atlases.org/. Researchers can easily apply Chinese2020 in their brain analysis tasks, such as SPM, by just replacing the default Caucasian template with Chinese2020 and viewing the results in Chinese2020 space by using Xjview^2^. A detailed instruction of how to use the template is shown in the Supplementary Material Section.

### Subjects and Experiments

Twenty-two healthy elderly subjects (10 males, 61.0 ± 6.1 years old) were recruited in this technology report. Subjects performed a simple finger tapping task. Data were acquired from a block design, alternating between self-initiated task (30 s) and rest conditions (30 s). During the task condition, subjects were instructed to generate a finger tapping at an interval of 3 s to 5 s. During the rest condition, subjects were instructed to relax and focus on the cross of the screen in front of them. For each subject, six task blocks and six rest blocks were acquired. The study was approved by the Clinical Research Ethics Committee of the Chinese University of Hong Kong (NTEC-CUHK ethics committee). Written informed consent was obtained prior to data collection from all of the participants.

### Image Acquisition

A 3.0 T Philips (Philips Achieva) scanner with an 8-channel SENSE head coil was used for data collection. For each subject, a high-resolution T1-weighted anatomical image was firstly acquired (repetition time (TR) = 7.54 ms, echo time (TE) = 3.52 ms, flip angle = 8°, voxel size = 1.04 × 1.04 × 0.6 mm^3^). Functional data were acquired by using a T2-weighted gradient echo-planar imaging sequence sensitive to blood oxygenation level dependent (BOLD) contrast (TR = 2500 ms, TE = 25 ms, flip angle = 90°, voxel size = 3 × 3 × 3 mm^3^). A total of 180 volumes of BOLD images were collected for each participant.

### Data Preprocessing

In this technology report, the functional MR imaging data were preprocessed using Statistical Parametric Mapping software (SPM8^3^). The preprocessing included discarding the first five volumes to allow for T1 equilibration, slice timing and realignment for rigid-body head motion correction. Participants who had head movement larger than 3 mm translation or 3° of rotation in any direction were considered as excessive head movement during scanning and were excluded. The T1-weighted anatomical image of each participant was then co-registered to the mean realigned functional images and subsequently segmented into GM, WM and CSF.

To compare the activation maps of using the SPM defaulted ICBM template and the Chinese2020 template, we segmented and normalized the anatomical and functional images to the ICBM template and the Chinese2020 template separately. Functional images were re-sampled to 3 × 3 × 3 mm^3^ and spatially smoothed with an 8 mm full-width half maximum (FWHM) Gaussian kernel.

Data were normalized for each subject separately by dividing the grand mean value of each session. Then a General Linear Model (GLM) was used to estimate the activity of the brain during the task. The onsets of tapping and instructions were entered into the model as different regressors. The regressors were convolved with the canonical hemodynamic response function (HRF). Additionally, six realignment parameters, CSF and WM signals were included as nuisance covariates. A high-pass filter (128 s cut off period) was used to remove low-frequency confounds, and a first-order autoregressive model was used to correct for serial correlations. The group-level analysis of one-sample *t*-test was performed to obtain the activation maps of the functional images normalized to the standard MNI space and the Chinese2020 space separately. The family-wise error rate (FWE) approach was used for multiple comparisons correction.

## Results

**Figure 1:**
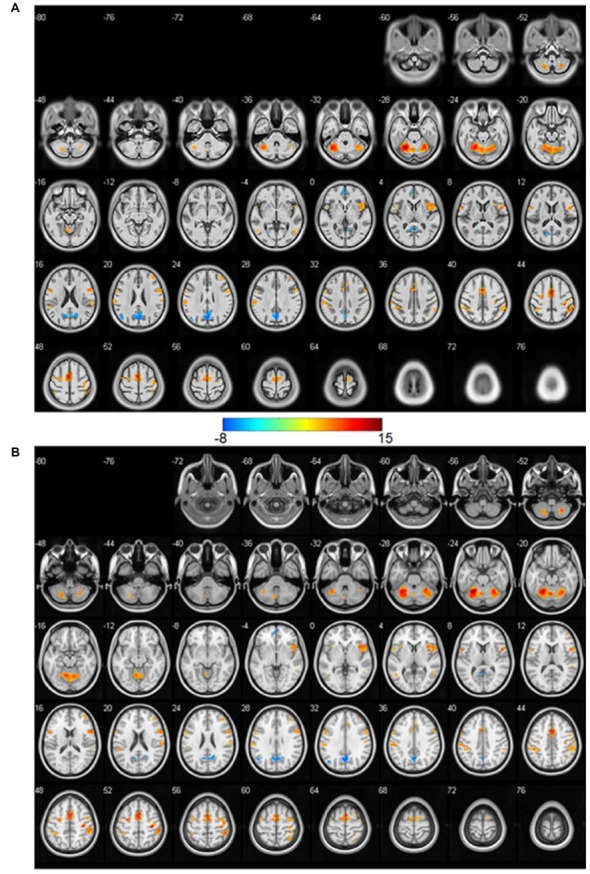
The significantly (*p* < 0.05, family-wise error rate (FWE) corrected, *T* > 6.45) activated *t*-maps based on the Chinese2020 template **(A)** and International Consortium for Brain Mapping (ICBM) template **(B)**. The color bar is in the middle, the positive activations are in warm color, and the negative ones are in cold color.

The *t*-maps of significant activations (*p* < 0.05, FWE corrected, *T* > 6.45) of Chinese2020 (A) and ICBM templates (B) were shown in Figure 1. We presented the whole brain coverage of each atlas to illustrate the size of the atlas in Figure 1. The finger motion activations detected by using Chinese2020 were similar to those of ICBM, both concentrated in the bilateral cerebellum, insula, middle temporal gyrus, and the motor cortex including primary, secondary and supplementary motor cortex. Both of the negative activations were located in the anterior cingulate cortex (ACC), and posterior cingulate cortex (PCC). The negative activated regions were the major hubs of the default mode network (DMN), which was expected since the DMN was supposed to be depressed during the self-initiated state.

**Figure 2:**
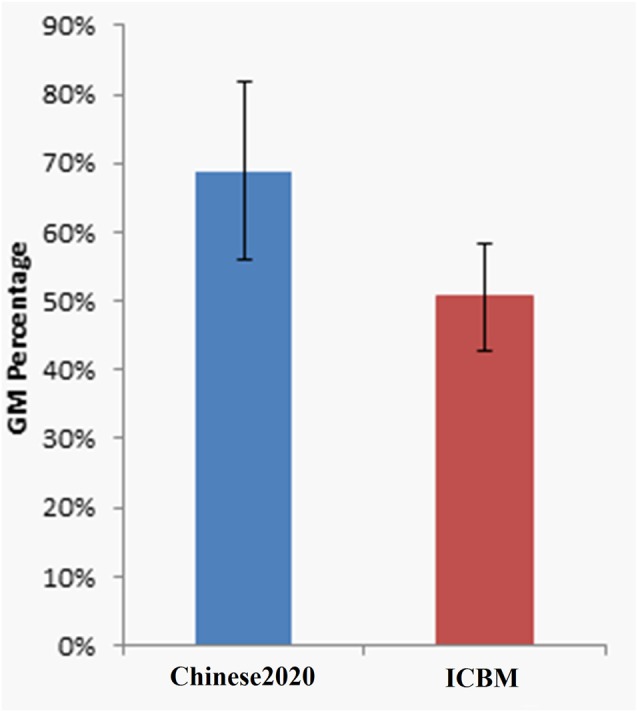
The GM percentage of activations based on Chinese2020 and ICBM were significantly different (*p* < 0.01).

In order to compare the effectiveness of each atlas quantitatively, the significantly activated clusters (*p* < 0.05, FWE corrected, cluster size >35) were summarized in Table 1. The first seven activated regions listed were the positive activations, and the last two regions of PCC and ACC were the negative activations. Since the activations were similar between the Chinese2020 and ICBM template, we showed the peak coordinates and the cluster size of the similar activated regions of the two templates alongside with each other. The cluster labels of these regions were presented in the rightmost column of the table. The size of positively activated clusters of the ICBM template was larger than those of Chinese2020 in the corresponding regions. Moreover, we further investigated the size of GM in each activated cluster (excluded cerebellum activations) to illustrate the alignment of the activations with the atlas. The mean of GM percentages of activated regions based on the Chinese2020 template (68.8 ± 12.9%) was significantly higher (*p* < 0.01) than those of the ICBM template (50.6 ± 7.7%). The comparison of GM percentages was shown in Figure 2.

**Table 1:** The summary of significant activation clusters by using the Chinese2020 and International Consortium for Brain Mapping (ICBM) templates.

	Chinese2020			ICBM		
	Peak coordinate	Cluster size	GM size	Peak coordinate	Cluster size	GM size	Cluster label
1	(−21, −51, −27)	663		(−21, −57, −21)	807		Cerebellum_L
2	(48, 6, 9)	196	148 (75.5%)	(51, 9, 6)	263	106 (40.3%)	Frontal_Inf_Oper_R
3	(−54, 6, 9)	89	68 (76.4%)	(−54, 6, 9)	155	73 (47.1%)	Frontal_Inf_Oper_L
4	(−60, −24, 30)	122	72 (59.2%)	(−45, −39, 45)	143	61 (42.7%)	Parietal_Inf_L
5	(54, −39, 45)	92	65 (70.7%)	(48, −42, 57)	173	96 (55.5%)	Parietal_Inf_R
6	(−3, 9, 42)	282	205 (72.7%)	(−3, 12, 45)	361	198 (54.9%)	Supp_Motor_Area_L
7	(33, −27, 42)	58	25 (43.1%)	(36, −24, 51)	113	63 (55.8%)	Postcentral_R
8	(−3, −66, 24)	201	135 (67.2%)	(0, −66, 33)	144	66 (45.8%)	PCC_L
9	(−3, 51, 0)	14	12 (85.7%)	(−9, 51, 0)	8	5 (62.5%)	ACC_L

## Discussion

In this technology report, we introduced the usage of the Chinese2020 template and demonstrated its effectiveness on an fMRI study based on Chinese populations. The Chinese2020 template was developed based on more than 2000 Chinese subjects, and the construction method of the template targeted on dealing with variances of signal-to-noise ratio (SNR) and image intensity from multiple centers. Our previous study (Liang et al., 2015) proved that fewer deformations were required to normalize the Chinese subjects’ brain to Chinese2020 than to ICBM, suggested that Chinese2020 represented the characteristics of Chinese populations better than ICBM. To further illustrate the effectiveness of Chinese2020 on functional neuroimaging analysis, a finger tapping fMRI study of Chinese populations was introduced in this technology report and the activation maps normalized by using Chinese2020 template and the ICBM template were compared.

From the statistical results of the finger tapping task, we could see that the activations based on Chinese2020 were aligned well with the gyrus of the cortex, and the major differences by using the two atlases (i.e., Chinese2020 and ICBM) may be due to the differences of morphometric and volumetric of the atlases. To further quantify the alignment of activations with GM, we calculated the percentage of activations located in GM in each cluster of the Chinese2020 and ICBM template, respectively. Because the activations were supposed to be located within GM of the cortex, we expect an atlas with higher specificity will naturally result in higher GM percentage within the activated clusters. The results demonstrated that the activations normalized to the Chinese2020 had significantly better alignment with GM than those of ICBM. Taken together, these results suggested that Chinese2020 provided a better template for Chinese populations than the standard ICBM template based on Caucasian populations.

In this technology report, the performance of Chinese2020 template on neuroimaging analysis was validated by using SPM in an fMRI study. Since the template is distributed in a commonly used NIFTI format, it could be flexibly used in other popular image processing packages, such as FSL and AFNI, as well as for DTI study. In further studies, we would like to investigate the effectiveness of the dynamic Chinese brain templates on the fMRI studies of various age ranges. In our previous study (Liang et al., 2015), we also developed a series of Chinese brain templates for 12 different age brackets (i.e., 20, 25, 30, 35, 40, 45, 50, 55, 60, 65, 70 and 75 years). Previous study has reported that age has a significant effect on brain structure and morphological characteristics (Kovalev et al., 2003). This has been further demonstrated in our previous study (Liang et al., 2015), and the template of age 30 would represent the group of young Chinese subjects better than the Chinese2020 template averaged from all ages. The investigation of different templates of various ages (or genders) will need a much larger dataset than that used in this technology report. We are looking forward to verify the effectiveness of the dynamic Chinese brain templates on other fMRI studies that could evoke the subcortical nuclei such as the limbic system and amygdala.

In conclusion, we introduced the usage of the Chinese2020 template in details, which could benefit large amounts of neuroimaging studies in Chinese populations. This technology report indicated that Chinese2020 would represent the characteristics of Chinese brains better than the ICBM one. We hope that the Chinese2020 template can contribute directly to the future brain imaging studies in Chinese populations.

## Author Contributions

LS and PL designed the study and experiments, contributed to drafting the main body of the manuscript. YL and KL contributed to data analysis and manuscript preparation. VCTM and WCWC contributed to interpretation and refined the manuscript. DW prepared the study design and data analysis. And KcL managed the subject recruitment.

## Conflict of Interest Statement

The authors declare that the research was conducted in the absence of any commercial or financial relationships that could be construed as a potential conflict of interest.

## Footnotes

^1^www.talairach.org/

^2^http://www.alivelearn.net/xjview8/

^3^http://www.fil.ion.ucl.ac.uk/spm/
