# Supplementary material for: Using Large-Scale Statistical Chinese Brain Template (Chinese2020) in Popular Neuroimage Analysis Toolkits
Source: Front Hum Neurosci. 2017 Aug 17;11:414. doi: 10.3389/fnhum.2017.00414 (PMC5562686; doi:10.3389/fnhum.2017.00414)
Supplement: Supplementary file 1 [file Data_Sheet_1.docx]

**Supplementary Material**

There are several varieties of Chinese2020 that could be downloaded, namely: the T1-weighted template, tissue probability templates and AAL atlas. The templates are distributed in NIFTI (“.nii”) format, and can be used in popular image processing packages, such as SPM (http://www.fil.ion.ucl.ac.uk/spm/), AFNI (https://afni.nimh.nih.gov/afni/), FSL (<http://fsl.fmrib.ox.ac.uk/fsl/fslwiki/>) and MRIcron (https://www.nitrc.org/projects/mricron). The T1-weighted template, grey matter (GM), white matter (WM), and cerebrospinal fluid (CSF) probability maps of the whole population are shown in **Figure S1**. Researchers can easily apply Chinese2020 in their brain analysis tasks by just replacing the default Caucasian template with Chinese2020and viewing the results in Chinese2020 space by using Xjview (http://www.alivelearn.net/xjview8/).


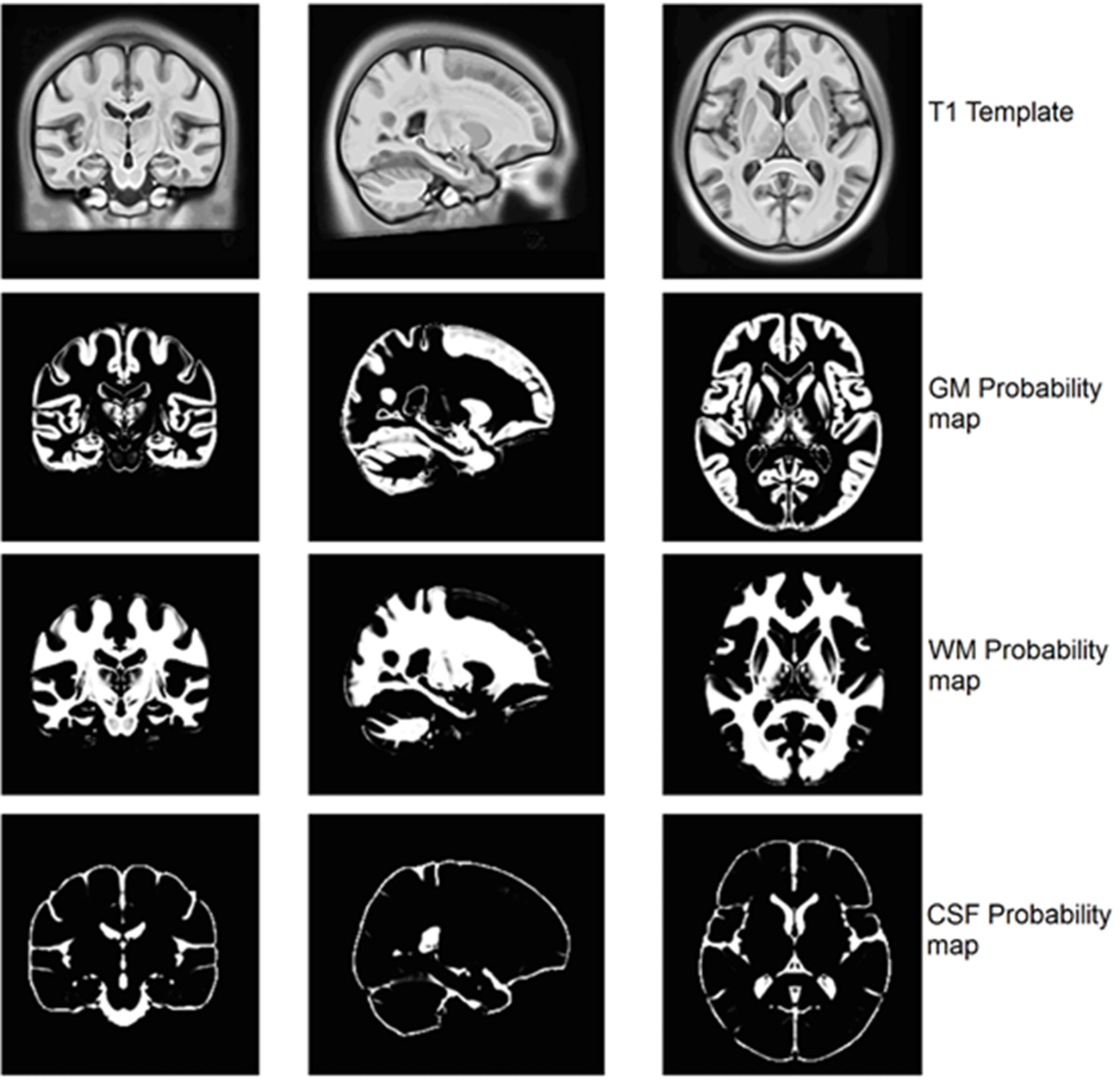


***Figure S1****. The T1 template, GM, WM and CSF probability maps of Chinese2020 are shown from the top row to the bottom.*

## **The Usage of Chinese2020 in SPM**

In SPM, a widely used approach to achieve image normalization is based on warping the tissue probability maps of the individual brain MRI data to the standard tissue probability maps. Therefore, the adoption of Chinese2020 in SPM is basically to replace the defaulted ICBM templates with the tissue probability maps of Chinese2020 during the segmentation. The operation procedure is as follows (**Figure S2**):

1. Launch SPM, and click the **Segment** icon.
2. Change the default tissue probability maps to those of Chinese2020.

Double click **Tissue probability maps**, unselect all the default probability maps, and select the Chinese2020 tissue probability maps with the order of “grey.nii”, “white.nii”, “csf.nii”.


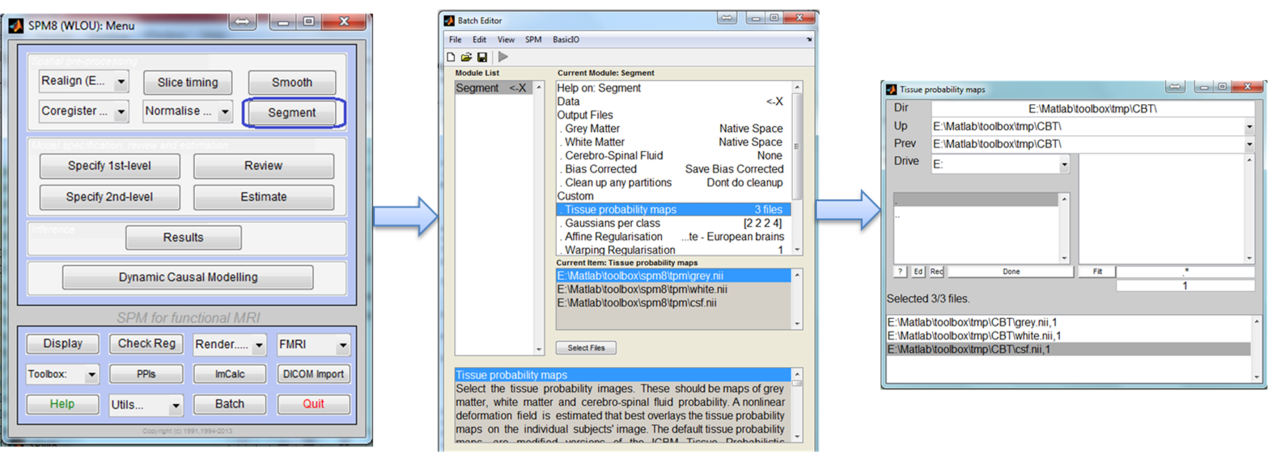


***Figure S2****. Flowchart of replacing the tissue probability maps of the ICBM template with those of the Chinese2020 template.*

## **Result viewing**

We recommend users of the Chinese2020 template to view the brain/results by utilizing the powerful viewing software “Xjview”. Before viewing the statistical maps overlaid on Chinese2020, several setups should be set up as follow.

1. Some files related to Chinese2020 and the size of atlas should be revised. The setups of Chinese2020 are noted in the “Readme.txt” file of the result viewing folder on our Chinese2020 website (<http://www.chinese-brain-atlases.org/>). Please download this folder.
2. After downloading the result viewing folder of Chinese2020, replace the “**TDdatabse.mat**” in the original Xjview folder with the Chinese2020 one. Then, replace the matrix T of “[onelinestructure, cellarraystructure] = **cuixuFindStructure**(mni, DB)” in the “**xjview.m”** file from original:

T = [ 2 0 0 -92

0 2 0 -128

0 0 2 -74

0 0 0 1];

to: T = [ -1 0 0 91

0 1 0 -122.3

0 0 1 -85.3

0 0 0 1];

1. Change the default T1 template in the upper right window of Xjview to the Chinese2020 T1 template, as marked in **Figure S3**.


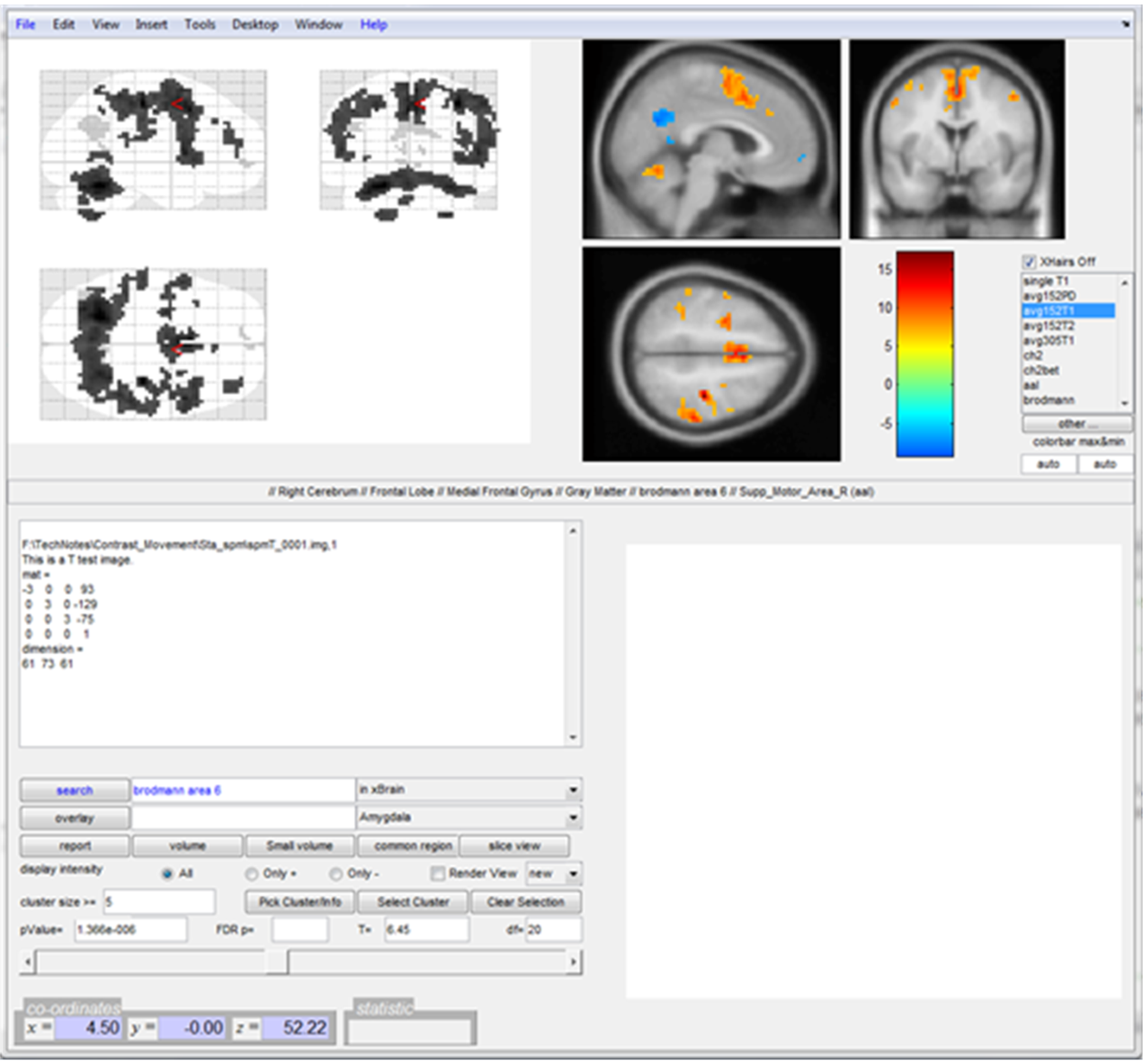


***Figure S3****. The graphical user interface (GUI) of Xjview. In result viewing of the statistical maps of activations, the underlaid template should be changed to the T1 template of Chinese2020. Click the button marked in the blue circle and select the T1 template of Chinese2020.*

**The coordinate conversion between MNI and Chinese2020 space**

The coordinates of Chinese2020 space are different from those of the MNI space. To report the coordinates of Chinese2020 comparable to other literature using the MNI space, we provide a coordinate converter (as shown in **Figure S4**) on our official website (<http://www.chinese-brain-atlases.org/>). If we input the **X, Y, Z** coordinates of Chinese2020 in the **Coordinate Transformation**, and click the button **sCBT-> MNI**, we could get the corresponding coordinates of the MNI space, and vice versa.


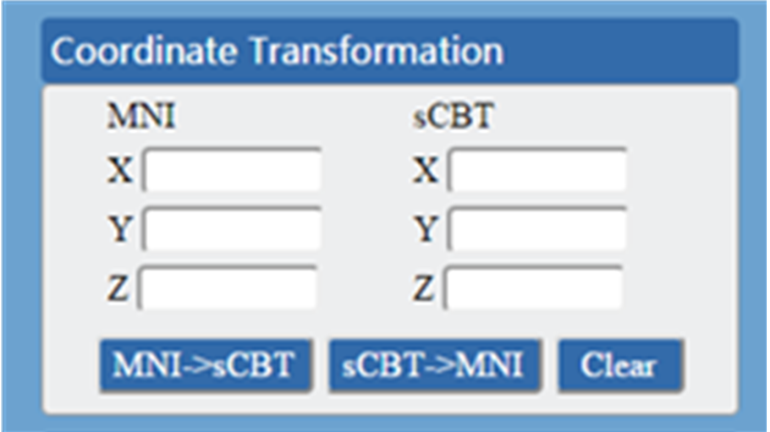


***Figure S4****. The coordinate conversion of the Chinese2020 space and the ICBM space is provided on the website of Chinese2020 (http://www.chinese-brain-atlases.org).*
